# Elastin-Based Materials: Promising Candidates for Cardiac Tissue Regeneration

**DOI:** 10.3389/fbioe.2020.00657

**Published:** 2020-06-30

**Authors:** Israel Gonzalez de Torre, Matilde Alonso, Jose-Carlos Rodriguez-Cabello

**Affiliations:** BIOFORGE, CIBER-BBN, Edificio LUCIA, University of Valladolid, Valladolid, Spain

**Keywords:** elastin-like, cardiovascular, cardiac patch, heart valve, vascular graft, angiogenesis

## Abstract

Stroke and cardiovascular episodes are still some of the most common diseases worldwide, causing millions of deaths and costing billions of Euros to healthcare systems. The use of new biomaterials with enhanced biological and physical properties has opened the door to new approaches in cardiovascular applications. Elastin-based materials are biomaterials with some of the most promising properties. Indeed, these biomaterials have started to yield good results in cardiovascular and angiogenesis applications. In this review, we explore the latest trends in elastin-derived materials for cardiac regeneration and the different possibilities that are being explored by researchers to regenerate an infarcted muscle and restore its normal function. Elastin-based materials can be processed in different manners to create injectable systems or hydrogel scaffolds that can be applied by simple injection or as patches to cover the damaged area and regenerate it. Such materials have been applied to directly regenerate the damaged cardiac muscle and to create complex structures, such as heart valves or new bio-stents that could help to restore the normal function of the heart or to minimize damage after a stroke. We will discuss the possibilities that elastin-based materials offer in cardiac tissue engineering, either alone or in combination with other biomaterials, in order to illustrate the wide range of options that are being explored. Moreover, although tremendous advances have been achieved with such elastin-based materials, there is still room for new approaches that could trigger advances in cardiac tissue regeneration.

## Introduction

Cardiovascular diseases (CVDs), which are a group of illnesses that directly affect the heart and its normal function, including valve failure, stroke, aneurism of coronary vessels and other heart diseases, are the leading cause of death in the United States and Europe. Indeed, in the period 2014–2015, the annual direct and indirect cost of CVDs in the United States was an estimated 351.2 billion dollars, including 213.8 billion in expenditures and 137.4 billion in lost future productivity attributed to premature CVD and stroke mortality (Benjamin et al., 2019).

The use of new biomaterials with enhanced biological and physical properties has opened the door to new approaches in cardiovascular applications. Biomaterials, as defined by Williams (1999), have been used for many different purposes in the field of regenerative medicine, especially as artificial extracellular matrixes, vehicles for cell delivery and controlled delivery systems for bioactive molecules. It could be thought that a special tissue, such as cardiac muscle may require biomaterials with special characteristics, although basically they should present biocompatibility, adequate mechanical strength, bioresorption or biodegradation, adequate internal morphology and cell-friendly fabrication (Ruvinov et al., 2014; Salerno and Netti, 2014), as is also the case for biomaterials used in other biomedical applications.

Elastin, which is an elastomeric, insoluble and fibrous protein, is one of the principal components of the extracellular matrix (ECM) and is mainly found in those tissues in which high deformations are required. Indeed, elastin can undergo hundreds of millions of cycles with no memory effect while maintaining its original behavior. In the cardiovascular field, it is of paramount importance in blood vessels, heart valves and other cardiac tissues (Powell et al., 1992; Scott and Vesely, 1995; Vesely, 1997; Huang et al., 2018). Elastin is necessary from a mechanical point of view and is also involved in many cell-signaling processes (Almine et al., 2010a, 2012). It is formed by the crosslinking of tropoelastin, its soluble precursor, in a process known as elastogenesis, which has been excellently described elsewhere (Hinek and Rabinovitch, 1994; Brown-Augsburger et al., 1995, 1996; Daamen et al., 2007; Almine et al., 2010b; Vindin et al., 2019). As such, tropoelastin itself (either natural or recombinantly produced) has been widely studied and applied in tissue engineering (Clarke et al., 2006; Mithieux et al., 2009; Wise and Weiss, 2009; Annabi et al., 2017). Unfortunately, once elastin has been degraded or disrupted, it is not easy to repair, at least without markedly affecting the natural alignment and arrangement of the undamaged tissue (Rodríguez-Cabello et al., 2018b). Moreover, its turnover is very low in healthy adults, with elastogenesis primarily occurring during the late fetal and early neonatal periods (Uitto and Larjava, 1991). As such, elastogenesis, which is a complicated process that has already been described elsewhere (Indik et al., 1989; Brown-Augsburger et al., 1997), decreases with age (Vrhovski and Weiss, 1998; Miriam et al., 2013), and is difficult to achieve under *in vitro* conditions (Flanagan et al., 2009; Syedain et al., 2011; Driessen-Mol et al., 2014; Moreira et al., 2016). Given this lack of elastogenesis in adults and the crucial importance of elastin in cardiac tissues, some researchers have focused their efforts on the use of elastin-based biomaterials, or at least those based on the repetition of the amino acid sequence valine-proline-glycine-valine-glycine (VPGVG) that codes for the elasticity of the elastin molecule according to Urry's model (Urry, 2006). These five amino acids form a β-turn, with proline-glycine at the angle of the turn and a 4 → 1 hydrogen bond connecting the keto group of the first valine to the amino group of the fourth valine along the sequence. A helical arrangement is produced by repetition of these β-turns to form a so-called β-spiral in which the β-turns act as spacers between the turns of the spirals. Valine-glycine sequences allow large-amplitude, low-frequency vibrations. Reductions in the amplitude of these vibrations cause a marked decrease in the entropy of the segment, thus providing the driving force for a return to the relaxed state, which drives the elastic behavior of the protein (Tamburro et al., 1990, 1992, 2003).

**Figure 1:**
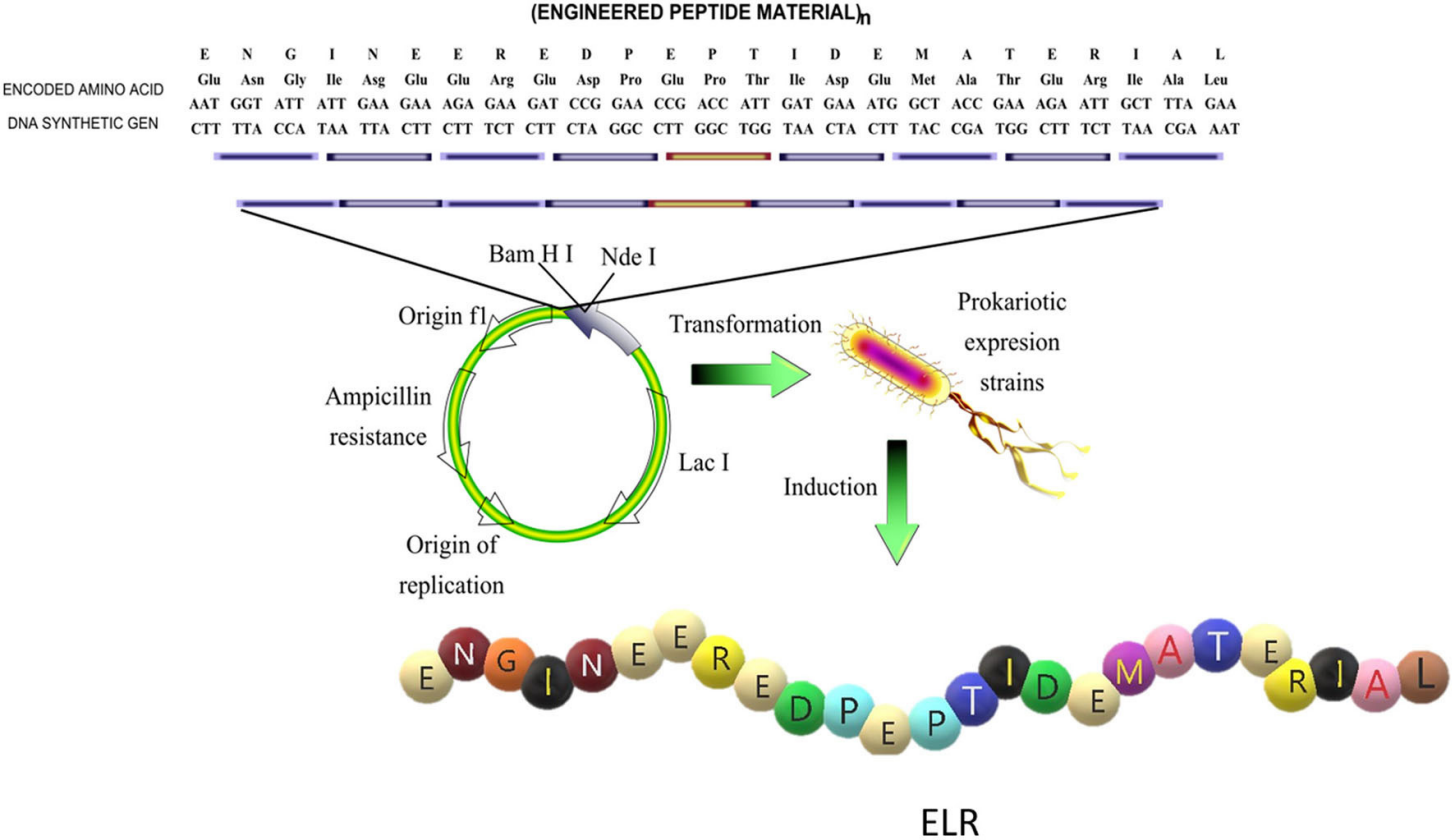
Schematic representation of the design of the sequence and production of an elastin like recombinamer (ELR). Reproduced with permission from Rodríguez-Cabello et al. (2018b).

Among these biomaterials, elastin-like polypeptides (ELPs) or elastin-like recombinamers (ELRs), depending on whether they are synthesized (Rodriguez-Cabello et al., 2017) by traditional chemical synthesis or by recombinant techniques, respectively, have been shown to be promising biocompatible candidates for use in tissue-engineering applications (Ibáñez-Fonseca et al., 2018). ELRs are based on the repetition of the five amino acids that confer the elastic properties to the elastin molecule, namely VPGVG, in which the V at the fourth position can be substituted by any amino acid except proline (Rodríguez-Cabello et al., 2018a). The use of recombinant techniques employing iterative-recursive methods ensures a high control over the amino acid sequence that forms the ELR and a very high monodispertsity, which is one of the principal problems of traditional chemical synthesis (Won and Barron, 2002; Chilkoti et al., 2006). This control allows the possibility to tailor ELRs with specific mechanical properties and include specific biofunctionalities for the target application or tissue (Figure 1).

ELRs exhibit a thermoresponsive behavior in aqueous solvents, which means that above a certain temperature, known as the transition temperature (T_t_) (Urry et al., 1992; Urry, 1997), they undergo a self-assembly process directed by electrostatic interactions that trigger a change in the conformation of the protein. This change consists of the exclusion of water, thus resulting in loss of the clathrate-like extended structure of the ELRs that leads to a viscoelastic state in which the governing structure is a β-spiral containing three units of the basic pentamer VPGVG, which form a type II β-turn per turn of the spiral, as mentioned. Previously. This change in conformation is accompanied by a shortening in the length of the ELR and release of a certain quantity of energy (Urry, 1993). The temperature (Tt) that defines this change is determined mainly by the amino acid sequence, the polarity of the amino acid present in the fourth position and the molecular mass of the ELR molecule (Urry, 1993).

Although one very optimistic goal in this field, at least currently, is to create a completely functional heart using tissue engineering, significant efforts are being dedicated to the three main sections into which this field can be subdivided, namely cardiac muscle, vasculature and heart valves (Zandonella, 2003; Sacks et al., 2009). In this review, we will focus on the latest results arising from the application of elastin-like materials in these three main areas.

## Cardiac Muscle Regeneration and Angiogenesis

It is very important to understand the special characteristics of cardiac tissue. This muscle is mainly formed by cardiomyocytes, a kind of hypertrophied muscle cell, which are perfectly aligned and electrically synchronized with the surrounding cells. As they are permanently beating, a constantly high intake of nutrients and oxygen is required to meet their metabolic needs. These nutrients and oxygen are supplied by a dense and very well-organized vascular network, in which smooth muscle cells and endothelial cells are the main cell sources that form and maintain the vascular network (Eng et al., 2016).

Acute myocardial infraction (AMI) has traditionally been defined as a decrease or suspension of blood flow to a portion of the cardiac muscle leading to necrosis. It usually results as a consequence of a blood clot in an epicardial artery that supplies nutrients and oxygen to that region. However, the definition of this kind of event has been changed recently—not all cases now unavoidably involve a blood clot—to a discrepancy between the amount of oxygen that the cardiac muscle requires and the supply that it effectively receives. This discrepancy may lead to myocardial damage, usually in the left ventricle (Saleh and Ambrose, 2018). As in any other muscle tissue, the subsequent ischemia produces a necrotic wound and a reduction in the ability of the affected region to perform its normal function. Therefore, in the case of cardiac muscle, this reduction could compromise the pumping capacity of the entire organ. From a histological point of view, slight changes can be appreciated after the MI. In the first 12 h, hypereosinophilic changes to the myocyte sarcoplasm are present before neutrophilic infiltrates. Subsequently, coagulative necrosis begins and progresses over the next 3 days, resulting in loss of myocyte nuclei and neutrophilic infiltration. Myocyte fragmentation and early phagocytosis occur during the first week. After that time, granulation tissue evolves to form a denser region of collagen which, over the following few weeks, will produce a poorly populated scar tissue with dense and compact collagen. All these events, accompanied by wall thinning and ventricular dilation, impede the normal functioning of the affected region (Pasotti et al., 2006; Ruvinov et al., 2008; Seliktar and Shapira-Schweitzer, 2010). As can be seen, the steps after MI follow a similar path to the wound-healing process (Stroncek and Reichert, 2008) until the final scar is formed, although they are aggravated by what is defined as “infarct extension.” This “infarct extension” is characterized by a remodeling of the ECM in both the directly damaged region and in the viable borders and beyond, reaching the remote region of the ventricle. The limitations arising due to the wound healing process in the affected region are importance as regards rehabilitation of the lost functionality in the affected region, and the limited ability of adult cardiomyocytes terminally differentiated after a myocardial infarction to regenerate clearly affects the regeneration of the affected region (Huang et al., 2018). To overcome this low regeneration ability of adult cardiomyocytes, numerous research groups have attempted to create cardiac patches and injectable systems.

Scaffolds used in cardiac tissue engineering have to provide both an adequate environment to promote cell growth and an integrative system that allows perfect conjunction with the surrounding tissues which, ideally, should be replaced by a perfectly functional natural tissue. Regardless of whether an acellular or cellular approach is used, such scaffolds (injected or preformed) have to provide support to create the vascular network needed to supply the nutrients and oxygen needed by the new cells that will populate them. This vascular network can be pre-formed *in vitro* or induced *in vivo* but, in both cases, has to be integrated and well-connected to the pre-existing natural vasculature from undamaged regions. As such, this is one of the key issues that biomaterial scientists have to overcome when creating engineered tissues that can survive in the long-term. Indeed, the lack of appropriate vascular integration is one of the key problems of some approaches for the creation of cardiac patches (Ruvinov et al., 2008; Zhang et al., 2018).

In light of the above, biomaterials for cardiac patches must be biocompatible and must provide the correct mechanical stiffness, matrix density, and viscoelasticity to induce angiogenesis together with the appropriate biological clues (Crosby and Zoldan, 2019). In this regard, ELR/ELP-based scaffolds can be created with tunable mechanical properties that can be adjusted on-demand, depending on the final application, simply by varying the final concentration and/or amino acid composition of the polypeptides. Scaffolds based on elastin-like materials with stiffnesses ranging from a few hundred to some tens of thousands of Pascals have been described in the literature (González De Torre et al., 2014; Gonzalez De Torre et al., 2016; De Torre et al., 2015; Cipriani et al., 2018), thus highlighting the mechanical versatility of this type of biomaterials. Moreover, although mechanical and physical features are critical for tissue-engineering applications, the micro-topography of elastin-derived materials has a crucial impact on the alignment and fate of cardiac cells. Thus, micropatterned tropoelastin-based hydrogels have been shown to promote CM attachment, spreading and elongation. These micropatterned scaffolds also maintained the phenotype and contractile properties of the CMs, and can be electrically stimulated to induce or control the beating of the CMs seeded on them (Annabi et al., 2013).

**Figure 2:**
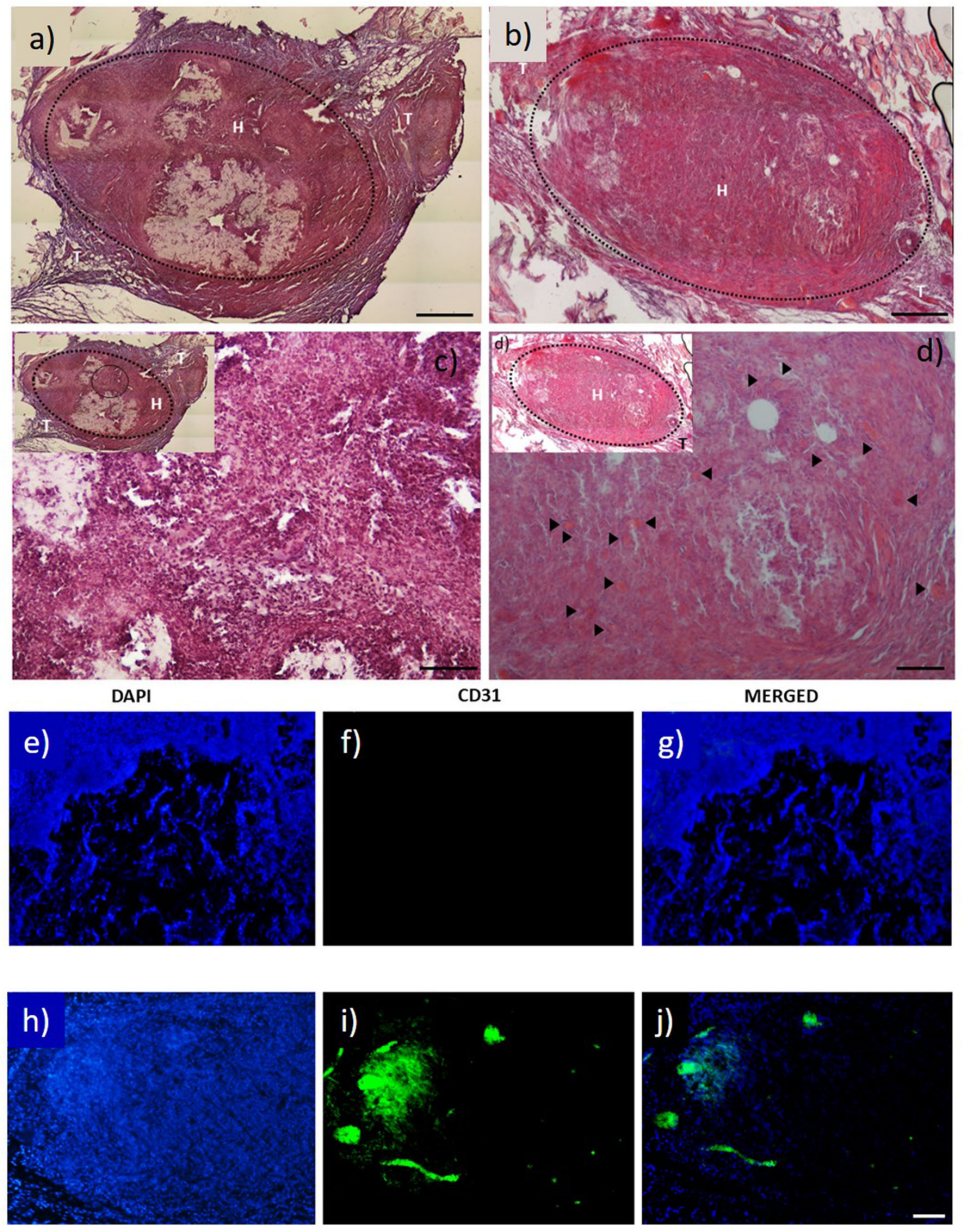
H&E staining images of: **(a)** ELR factor-free hydrogel; and **(b)** ELR-QK hydrogel; **(c,d)** magnification 20× of image and **(b)**, respectively. Black arrows indicate the presence of newly formed capillaries. Scale bar **(a–d)** images 100 μm. **(e–j)** CD31 immunofluorescent staining images: **(e–g)** factor free ELR hydrogel (ELR), **(h–j)** ELR-QK hydrogel. DAPI: nucleus staining **(e,h)**. CD31: expression of CD31 protein **(f,i)**. MERGED: merged channels (DAPI + CD31) **(g,j)**. Scale bar: 50 μm. Reproduced with permission from Flora et al. (2019a).

Elastin-derived materials have proved to be suitable biomaterials for the creation of scaffolds that can trigger angiogenesis. Thus, Robinet et al. have shown that peptides containing the sequence VGVAPG (found in tropoelastin and elastin-like materials) can trigger the angiogenic phenotype of ECs from the micro- and macrovasculatrue (Robinet et al., 2005). This was corroborated by Reddel et al., who showed that exposed elastin sequences are able to increase the levels of matrix metalloproteinases (MMP-2), which contribute to the digestion of connective tissue growth factor (CTGF) and, together with transforming growth factor (TGF-β1), increase the amount of free vascular endothelial growth factor (VEGF) (Reddel et al., 2013), both of which are closely related to angiogenesis. An option to increase the angiogenic potential of the ELRs is to fuse them with this grow factor (VEGF). the Three VEGF genes, namely VEGF-1, VEGF-2 or VEGF-C and VEGF-3 or VEGF-B, VEGF-D, VEGF-E, have been identified in humans (Seandel et al., 2001; Vale et al., 2001). Although located in different chromosomes, all these genes share similar features and behaviors but have different actions in endothelial cells (ECs), affecting different endothelial-specific fms-like tyrosine kinases (VEGFR-1, VEGFR-2, VEGFR-3) and therefore producing different effects in ECs. For instance, VEGFR-1 is related to the assembly of ECs into tubes to yield functional vessels (Seandel et al., 2001), VEGFR-2 is involved in EC proliferation and migration (Shalaby et al., 1995; Carmeliet and Collen, 1997), while VEGFR-3 is more closely related to the lymphatic system (Jeltsch et al., 1997). VEGF can be incorporated into elastin-like scaffolds to provide a pro-angiogenic environment to increase the inherent angiogenic potential of the elastin-derived materials (Flora et al., 2019a) (Figure 2). Moreover, in some studies not related with the regeneration of the cardiac muscle but very related with vascular diseases, as for instance renovascular disease, this combination of ELR-VEGF have been tested in order to increase its angiogenic potential. Both, *in vitro*, and more importantly, *in vivo* experiments showed that this biomaterial was able to stimulate the cells related with vessel formation to produce tubular structures. Accumulations of ELRs-VEGF in stenotic kidneys triggered the newly formation of micro-vessels in the cortical and medullar area of the kidneys. ELRs-VEGF did not alter the potency of VEGF alone but this combination increased the half-life with respect to free VEGF resulting in a more effective at recovering the renal function (Chade et al., 2016). However, despite the evident angiogenic potential resulting from the incorporation of VEGFs into scaffolds for tissue-engineering applications (Testa et al., 2008), some studies have pointed out that the use of growth factors has some intrinsic drawbacks, such as low stability and, subsequently, a loss of bioactivity, as well as the possibility of immunogenicity (Lee et al., 2011). To overcome these possible disadvantages, synthetic peptides known as QK peptides, based on the 17–25 alpha region of VEGF_165_ that binds to the main VEGF receptors, such as VEGFR-1 and VEGFR-2, have been designed (D'Andrea et al., 2005). This QK peptide has shown excellent stability while maintaining its helical structure and bioactivity (Diana et al., 2008; Leslie-Barbick et al., 2011). QK peptides have been incorporated into ELRs in order to enhance their angiogenetic ability with the aim of obtaining better integration of the tissue-engineered scaffold into the natural surrounding tissue. An initial *in vitro* study showed that QK peptides anchored to the backbone of ELP-based hydrogels could enhance the adhesion and proliferation of HUVECs in 2D systems and could stimulate more sprouting in 3D environments (Cai et al., 2014). In a similar study, Flora et al. demonstrated that tethering QK to ELRs provided a proangiogenic environment in a similar manner to VEGF and that this ELR-QK combination enhanced the formation of new functional capillaries *in vivo*. This new microvasculature is essential for the survival of the cells colonizing the tissue-engineered scaffold, thus suggesting that this approach could be of great interest in a wide-range of cardiovascular applications that require the spatial-temporal control of growth factors (Flora et al., 2019a). Under the light of the previous lines, the inclusion of GFs into ELR based hydrogels for cardiac regeneration or even fused with the ELR molecules could be an interesting option. However, the use of free GFs entrapped on ELR based hydrogels could have some limitations, as for instance low effective time before their (GFs) degradation. Other key factor to be considered is the dose and how to obtain the optimal concentration from a GF loaded hydrogel. Such spatial-temporal control is still a great challenge even though many strategies have been followed (Martino et al., 2014, 2015). In the case of GFs fused to ELRs, the latency of the GF is clearly increased, as previously mentioned. Moreover, it has to be considered when designing a scaffold based on ELRs fused with GFs, that an increase on the half-life of the GF could be reflected in a lowering of the dose to avoid overdose issues.

In addition to the intrinsic benefits of elastin-derived materials, their tunable mechanical properties and the possibility of tethering pro-angiogenic factors, the option of incorporating selected bioactivities into the backbone of ELRs is essential to meet the precise requirements of specific applications. In this case, cardiac tissue requires a highly vascularized environment, therefore a precise combination of bioactive sequences that could trigger a specific response of endothelial cells compared with other cell types is of great interest. In this regard, Flora et al. have demonstrated that a correct choice of bioactivities (RGD and REDV, Arg-Gly-Asp, and Arg-Glu-Asp-Val, respectively), and the right proportion between them, could induce the selective adhesion of endothelial cells from a co-culture with human foreskin fibroblast (HFF-1) and human umbilical vein endothelial cells (HUVECs). Although the choice of a highly selective bioactive sequence for endothelial cells, such as REDV (Massia and Hubbell, 1992; Heilshorn et al., 2003), may not be sufficient in terms of adhesion and proliferation, in conjugation with other bioactive sequences this adhesion and proliferation can be enhanced without losing selectivity (Flora et al., 2018). In addition, the correct selection of bioactivities in terms of cell adhesion and proliferation properties is important. Thus, an appropriate degradation rate is paramount to synchronize the degradation rate of the scaffold and the regeneration rate of the damaged tissue. In this regard, a combination of protease-sensitive bioactive sequences that could harmonize the colonization rate of the cells and the degradation rate of the scaffold, or a cell-demanding degradable scaffold, could be the key to achieving complete and efficient regeneration with adequate vascularization (Trappmann et al., 2017).

**Figure 3:**
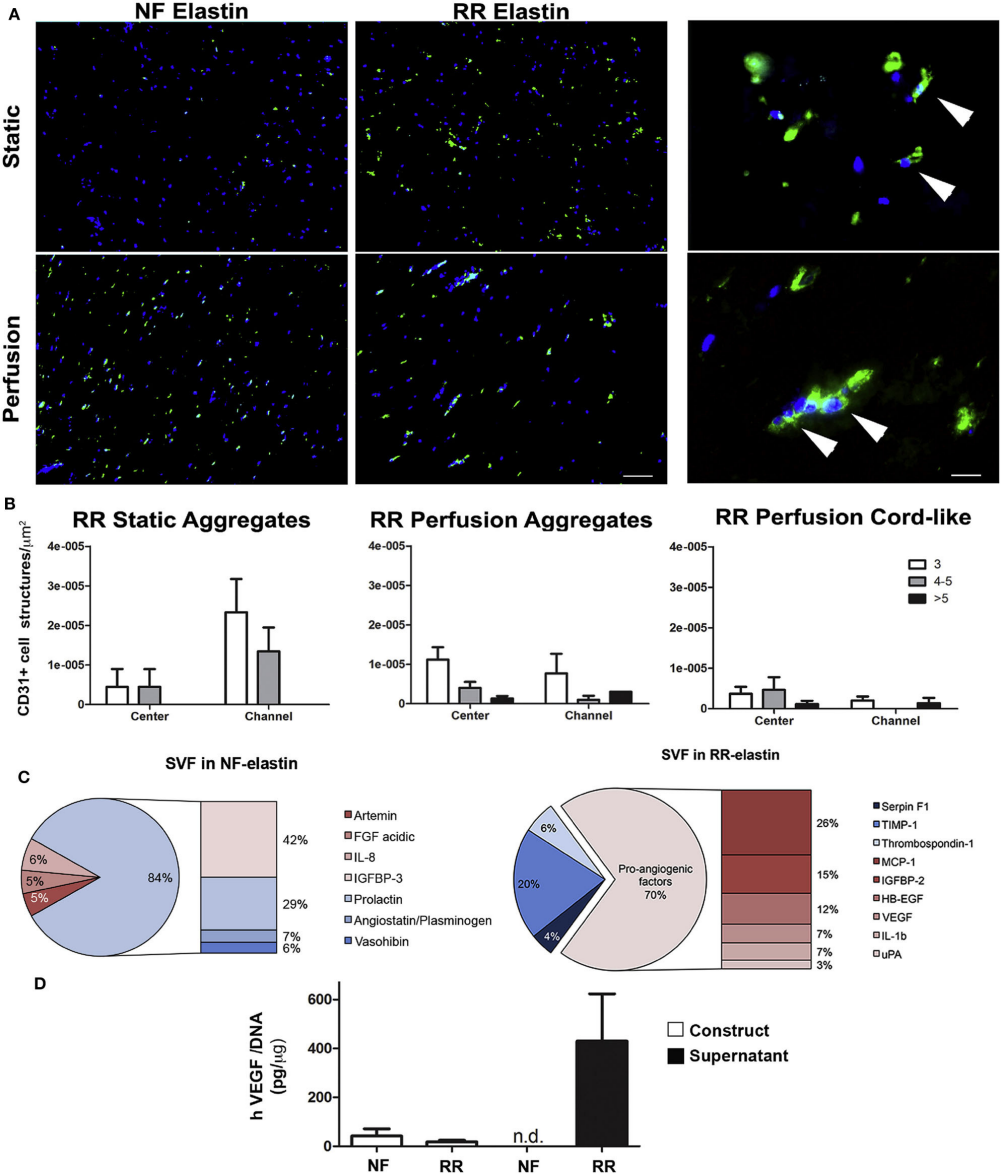
*In vitro* angiogenic potential of SVF cell-based ELR constructs. **(A)** Immunofluorescence staining for CD31 (green) of constructs generated by SVF cells in either non-functionalized (NF) or functionalized with REDV-RGD (RR-ELR) in static or perfusion-based culture at low (left) and high (right) magnifications. Nuclei were stained with DAPI (blue). High magnifications were provided only for the RR-ELR condition to distinguish between the formation of cell aggregates and cord-like structures. White arrowheads indicate the specific staining for endothelial cells. Scale bars = 100 and 20 mm, for low and high magnification, respectively. **(B)** Quantification of CD31^+^ cell aggregates formed in static or perfusion culture and cord-like structures in perfusion in areas proximal (channel) or distal (center) to the perfusion channels. Both aggregates and cord-like structures were distinguished by the number of endothelial cells included in them (3, 4, or 5, more than 5). **(C)** Pie graph representing all the upregulated factors in NF (left) or RR- (right) ELR constructs generated in perfusion culture, divided into pro- and anti-angiogenic factors. **(D)** Quantification of VEGF released by and entrapped in constructs generated in NF or RR-ELR in dynamic condition. Number of donors = 2 (duplicate samples for each donor) **(B–D)**. Reproduced with permission from Staubli et al. (2017).

Some successful attempts to introduce protease-sensitive sequences into elastin-like materials in order to modulate vascularization of tissue-engineered scaffolds *in vivo* have been performed. Thus, Staulbi et al. created a scaffold based on two different ELRs that combined cell-adhesion sequences, such as RGDs (general cell-adhesion sequence) and REDV (EC-specific cell-adhesion sequence) with an elastase-sensitive sequence, such as Val-Gly-Val-Ala-Pro-Gly (VGVAPG). A non-biofunctionalized ELR was used as control. These ELRs were chemically modified to bear chemically reactive groups that were used to create hydrogels under different conditions. The study aimed to evaluate the contribution of the gel structure and biofunctionalization on control of the angiogenic potential of this kind of material for use in cardiovascular applications. Stromal vascular fraction cells (SVF), a heterogeneous mixture of cells in which mesenchymal and endothelial/mural progenitor cells are the key cell types, were used in this study. To investigate the angiogenic potential of these scaffolds, several pro- and anti-angiogenic factors secreted by the SVF were evaluated *in vitro*. Scaffolds containing RGD, REDV, and VGVAPG sequences were prone to inducing cells to produce pro-angiogenic factors, such as monocyte chemoattractant protein-1 (MCP-1), insulin growth factor binding protein-2 (IGFBP-2), heparin-binding epidermal growth factor (HB-EGF), VEGF, interleukin-1b (IL-1b), and urokinase-type plasminogen activator (uPA). In contrast, with non-functionalized scaffolds, anti-angiogenic factors, such as insulin growth factor binding protein-3 (IGFBP-3), prolactin, angiostatin, or vasohibin, were mainly secreted by cells. Moreover, functionalized scaffolds showed higher endothelial cell infiltration than their non-functionalized counterparts. These findings were in agreement with those found in *in vivo* experiments, which showed a higher cell infiltration and better integration of the scaffold in functionalized scaffolds (Staubli et al., 2017) (Figure 3). As such, it can be concluded from this study that angiogenesis and host integration of these kinds of ELR-based scaffolds can be controlled by modulating the functionalization of the hydrogel alone, which may prove to be an excellent tool in cardiac tissue regeneration.

Other protease-sensitive sequences have been tested in ELRs to induce controlled biodegradability of the scaffolds and promote directed and guided cell infiltration. These sequences, namely YAVTG**DRIR**SASPASSA and YAVTG**GTAR**SASPASSA (DRIR and GTAR, respectively), were based on the previously mentioned urokinase plasminogen activator (uPA) (Straky and Heilshorn, 2009). These two sequences were combined with the previously mentioned general cell-adhesion sequence RGD to create two different kinds of scaffold, namely one with a slow degradation rate containing the DRIR sequence and one with a fast degradation rate containing the GTAR sequence. *In vitro* experiments showed that the different degradation rates depended on the bioactive sequence selected. However, the appearance of new functional vascular vessels within the regenerated region is more relevant from a cardiac tissue-engineering point of view as this allows the creation of a quickly colonizable scaffold. This finding suggests that such a fast degradability induces a fast colonization that is supported by the creation of new functional vasculature (Flora et al., 2019b), which could be crucial for a rapid and functional regeneration of the damaged cardiac tissue.

## Vascular Grafts (VGs)

The mismatch between the oxygen requirements of cardiac tissue and the amount that effectively reaches it is often caused by a failure in the blood supply due to the blockage or rupture of a blood vessel that must provide this oxygen. The first attempts at blood vessel substitution date back to the 1950s (Blakemore et al., 1952; Blakemore and Voorhees, 1954; De Bakey et al., 1958). However, in those days, the two main approaches for replacing damaged blood vessels, which are currently still the most widely used in surgical procedures, involved the use of an autologous (Brescia et al., 1966) or a synthetic graft (De Bakey et al., 1958). It is commonly accepted that the use of autologous vessels as grafts for bypass procedures is the best option even though limitations related to the availability of suitable autologous grafts could represent a problem. On some occasions, surgeons have to choose the synthetic route. However, this can lead to issues related to the small diameter of synthetic conduits, which have a particular propensity to re-stenosis and thrombus formation. Given these limitations, the field of vascular grafts is still relatively underdeveloped and new materials, concepts and approaches are being developed by materials and tissue engineering scientists in their search for the optimal conduit that fulfills all the requirements of this demanding application. Among the different options being studied, the use of tissue-engineered vascular grafts (TEVGs) is one of the most promising approaches to achieve the desired goal of a fully functional vascular vessel with a performance and biological properties similar to native ones. The leading principle in the field of tissue engineering for the creation of TEVGs is biomimicry. It is well-known that nature has served as the inspiration to recreate different structures, tools and devices (airplanes, helicopters, submarines, etc.). The same biomimicry, or imitation of nature, forms the basis for the generation of TEVGs. These TEVGs are intended to be more than mere tubes or conduits to channel blood without leaking as they must avoid immune rejection after implantation and should trigger the formation of autologous tissue that finally replaces the artificial implanted structure, thus forming a completely functional vessel. Basically, they have to accomplish a series of requirements or attributes, as discussed by Benrashid et al. in a nice review of TEVGs (Benrashid et al., 2016):

- Low risk of infectious complication
- Lack of an immune response
- Long-term patency
- Controlled degradation
- Suture integrity
- Suturability
- Ability to be remodeled by cells as a functional tissue
- Responsive to physiological stimuli
- Antithrombotic
- Internal structure that allows for cell migration/seeding
- Durability and mechanical strength
- Cost effectiveness
- Ready availability for use

The first TEVG was reported in 1986 and had a structure based on a multilayer collagen tube and a combination of endothelial and smooth muscle cells (SMCs) (Weinberg and Bell, 1986). Since this first approach, several other TEVGs have been created by varying the base biomaterial and/or cell type and source (Kannan et al., 2005; Buttafoco et al., 2006b; L'Heureux et al., 2006, 2007; Wang et al., 2007; Allen et al., 2014; Wystrychowski et al., 2014).

There are several fabrication techniques to obtain prosthetic VGs. Although it is not intended in this review to explore depply the different fabricating technologies to obtain prosthetic VGs (Wang et al., 2019). It is particularly interesting a novel method developed by Wang et al. in which sacrificial ice scaffolds are created by 3D printing and coated with different materials (tropoelastin alone or mixed with PCL or PDMS, and pure silk or silk/PMDS mixtures) to finally obtain scaffolds with the desired architecture after melting the inner ice core (Hiob et al., 2017).

Many synthetic plastic-based VGs have been designed and tested. Although all of these systems have different mechanical and biological properties, none of them are extensively used as substitutes for blood vessels in clinical practice (Liu et al., 2018). As such, we will not analyze them in this review as our focus here is on approaches that involve the presence of elastin or elastin-derived materials.

Elastin, which is one of the main components of the extracellular matrix in blood vessels, provides long-range deformability and resilience to these vessels, thus allowing a perfect adaptation and performance of the vessel to physiological demands. Elastin is a very stable molecule that rarely undergoes remodeling and exhibits high resistance to the action of degrading enzymes, with only elastase and metalloproteinases (MMPs) having some activity against this resistant molecule (Fornieri et al., 1992; Kielty et al., 2002; Chuang et al., 2009). Since elastogenesis is a process that rarely occurs *in vitro* (Hinderer et al., 2015), some approaches for generating TEVGs have focused their efforts on the use of elastin-derived materials to create these artificial blood vessels. One of the first such approaches involved decellularize and the removal of collagen from porcine arteries. The resulting structure, which mainly comprised the remaining elastin, was further stabilized by the use of penta-galloyl glucose (PGG). The stabilized constructs showed adequate mechanical properties and allowed cell repopulation of the scaffolds. *In vivo* experiments indicated a low calcification of the grafts (Chuang et al., 2009). This pathway, based on decellularized tissue scaffolds, has been widely studied, mainly starting from porcine tissues (Roy et al., 2005; Tillman et al., 2012; Syedain et al., 2014; Koobatian et al., 2016). Although all of them confirmed the feasibility of using TEVGs from decellularized animal vessels and their stable geometry, low thrombogenicity, and durable wall structure under *in vitro* and *in vivo* conditions none of them are being used clinically.

A second approach for obtaining tissue-engineered blood vessels (TEBVs) with some quantity of elastin in their structure involves the use of fibroblasts to create cell sheets that are subsequently rolled to obtain tubular constructs. These constructs are then dehydrated and decellularized to give a cylindrical structure comprising the extracellular matrix produced by the fibroblast. This acellular tubular substrate has two functions, namely as a barrier to avoid cell migration toward the lumen and as a substrate for endothelial cell seeding in order to obtain a complete endothelial layer covering the lumen of the tubular scaffold and mimicking the natural structure of blood vessels. This approach was developed by L'Heureux et al. (2006), who obtained TEBVs with two different internal diameters (4.5 and 1.5 mm) and a wall thickness of between 0.2 and 0.45 mm. These TEBVs showed good mechanical properties and performance and were found to integrate perfectly with the host vessel, at an anastomosis level, in *in vivo* experiments in primates. Unfortunately, the time required to create one of these TEBVs (more than 7 months) is too long to allow them to be used in emergency situations, such as stroke or aneurism.

Buijtenhuijs et al. prepared tubular constructs from a dilute acetic acid suspension of collagen I and elastin. These tubular constructs were tested *in vitro* by seeding smooth muscle cells, which were able to proliferate and colonize the scaffolds (Buijtenhuijs et al., 2004; Buttafoco et al., 2006a; Engbers-Buijtenhuijs et al., 2006). Similarly, combinations of different types of collagen and elastin cross-linked with glutaraldehyde scaffolds have been tested by several authors (van Wachem et al., 1994; Stitzel et al., 2006), although the presence of organic chemicals compromises the biocompatibility of the resulting scaffolds (Daamen et al., 2007). Electrospinning has been explored to process mixtures of collagen and elastin using poly(ethylene) oxide and NaCl as co-reagents. *In vitro* tests showed the proliferation of smooth muscle cells on these tubular scaffolds (Huang et al., 2001; Buttafoco et al., 2006b). Following on from the use of electrospinning, McKenna et al. developed a tubular vascular scaffold by dissolving recombinant tropoelastin in 1,1,1,3,3,3-hexafluoro-2-propanol and cross-linking with diusccinimidyl suberate. The resulting vascular scaffolds could be precisely designed in terms of length and diameter and exhibited adequate mechanical properties similar to those of native elastin, thus meaning that the use of organic solvents did not produce significant changes in the conformation of the recombinant tropoelastin. However, the presence of these organic compounds has to be controlled and limited to low values in order to avoid cytotoxic effects (McKenna et al., 2012).

Tropoelastin has been used as both a structural biomaterial for constructing VGs, as discussed above, and as an anti thrombogenic coating for VGs created from other materials. Thus, Sugiura et al. developed a bioresorbable VG made from a non-woven poly (glycolic acid) (PGA) fiber mesh coated with a copolymer sealant solution of poly(L-lac tic-co-e-caprolactone) (PLCL) and reinforced by electrospinning poly(L-lactic acid) (PLA) nano-fiber onto the outer side of the PLCL scaffold. This scaffold was functionalized with a monolayer of physisorbed tropoelastin molecules. These VGs were implanted in the infra-renal aortic position in mice and were shown to be able to control the proliferation of vascular SMCs, thereby reducing intimal hyperplasia. However, these authors pointed out that more acute thrombosis was found in the tropoelastin-treated group and that this could be attributed to occasional tropoelastin aggregation increasing the roughness of the luminal surface (Sugiura et al., 2017). On the contrary, some studies point that tropoelastin coated scaffolds inhibit intimal hyperplasia enhancing attachment and proliferation of endothelial cells and substantial reduction in platelet attachment (Wise et al., 2011; Kondyurina et al., 2017; Sugiura et al., 2017). Tropoelastin can be used to create electrospun vascular grafts and it seems that although tropoelastin retains its structural integrity mimicking the ECM and supporting cell growth, pure tropoelastin scaffolds lack of the sufficient tensile strength to be used as scaffolds for VGs (Wise et al., 2011; Hiob et al., 2017).

ELRs (or ELPs, depending their recombinant or chemical origin; Rodriguez-Cabello et al., 2017) are another large family of elastin-based materials that have been used to create VGs. In 2010, for example, Caves et al. published a study in which the procedure for manufacturing VGs from elastin-like proteins reinforced with collagen microfibers was described. This study showed how differences in fiber orientation, spacing and fraction affected the final physical and mechanical properties (burst pressure, compliance and suture retention) of the ELP-based VGs (Caves et al., 2010). ELRs alone have been used as biomaterials for electrospinning with the aim of forming an elastic and resistant mesh that can be used for tissue-engineering applications (Fernández-Colino et al., 2018; González de Torre et al., 2018) and, more specifically, for cardiovascular applications (Putzu et al., 2016, 2019). González de Torre et al. were the first to synthesize stable electrospun ELR fibers without the need for any additional crosslinking step after the electrospinning process. The scaffolds obtained showed a maximum percentage strain of 247.5 ± 36.08%, with a mean Young's modulus of 1.73 ± 0.95 MPa and excellent cytocompatibility in *in vitro* experiments. Similarly, Putzu et al. created tubular scaffolds from electrospun ELRs using a rotating mandrel as collector. The engineered VGs were formed by an inner layer mainly comprising ELRs containing REDV sequences (tetrapeptide specifically recognized by the integrin α4β1 present in endothelial cells) in order to selectively recruit endothelial precursor cells. The outer layers mainly comprised ERLs containing the general cell adhesion sequence RGD to induce the attachment of structural cells that could colonize the external part of the scaffold. The tubular scaffolds were cross-linked with 0.1% genipin/acetone solutions and subsequenty rinsed with PBS in order to eliminate any organic residues that could have cytotoxic effects (Putzu et al., 2016). In order to avoid the use of potentially cytotoxic crosslinking agents, the same group tried to electrospin a Silk-ELR copolymer containing amino acid sequences from silk (GAGAGS) and from elastin (VPGVG). This latter sequence was intended to provide elasticity and biocompatibility, while the silk sequence spontaneously self-assembled into β-sheets stabilized by hydrogen bonding. This combination was used to cover stents and catheter balloons to create fibrous scaffolds.

**Figure 4:**
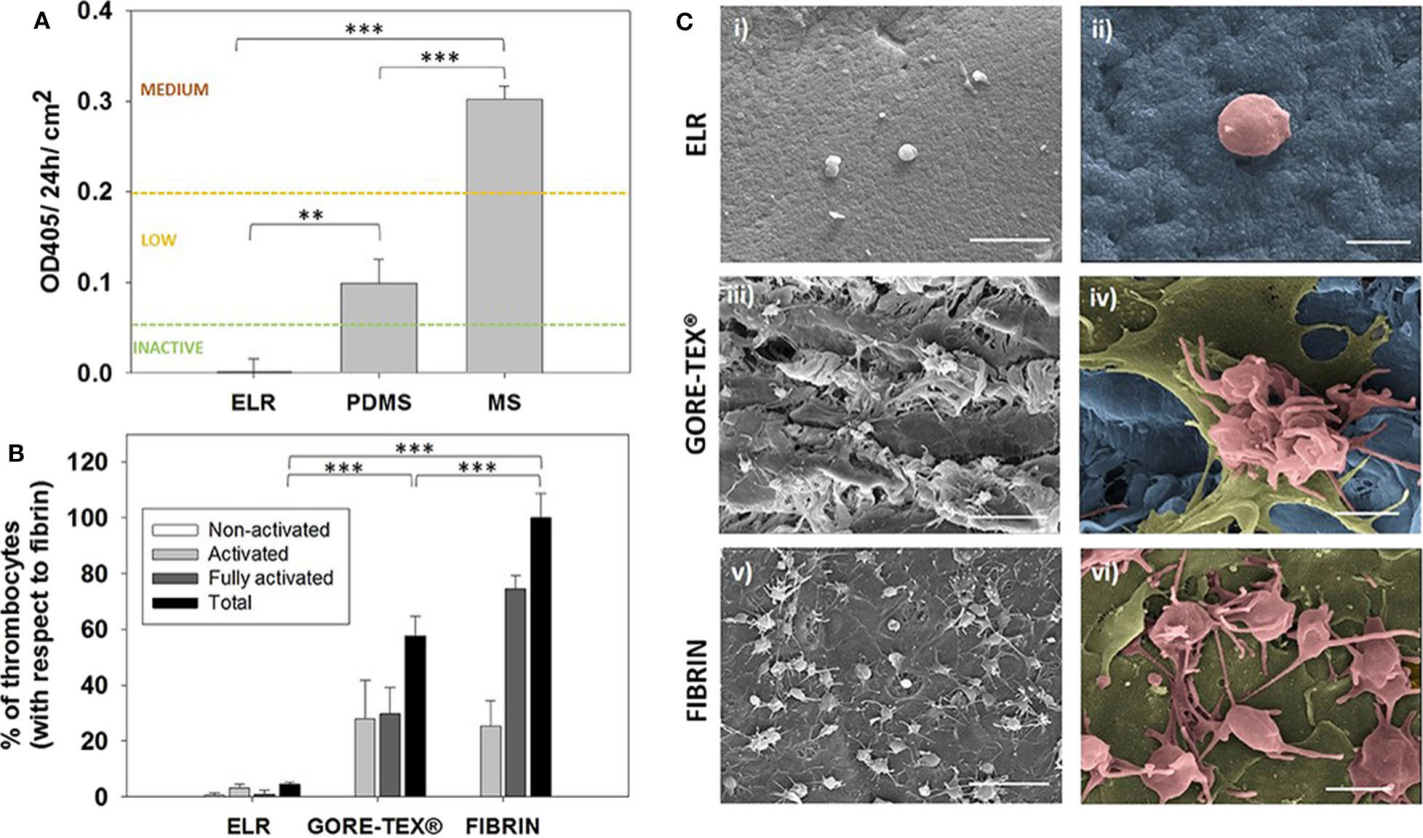
Hemocompatibility assessment. **(A)** Hemocompatibility of ELR-gels assessed using the C5 complement convertase activity. Control materials used for comparison: medical steel (MS) and polydimethylsiloxane (PDMS). **(B)** Evaluation of adhered platelets based on SEM images. Results are plotted as the mean of three different experiments ± standard deviation. **(C)** SEM images of (i,ii) ELR hydrogel, (iii,iv) GORE-TEX®, and (v,vi) fibrin-gel after 1 h in contact with platelet rich plasma. Activated and fully activated platelets were covering fibrin and GORE-TEX® surfaces, while ELR surfaces presented minimal platelet adhesion. In the images ii, iv, and vi, the ELR and GORE-TEX® scaffolds were colored in blue, the platelets with a spread morphology in gold and the more rounded platelets in salmon. Scale bars: (i,iii,v): 10 μm; (ii,iv,vi) 2 μm. Reproduced with permission from Fernández-Colino et al. (2018). **p < 0.01, ***p < 0.001.

Recently, Fernandez-Colino et al. developed a small caliber multicomponent vascular substitute based on a combination of three components, namely a textile reinforcement, a macroporous ELR-based scaffold and an external layer of electrospun PCL. These multicomponent vascular grafts exhibited good mechanical properties in terms of burst pressure (up to 2,000 mmHg) and suture retention force (higher than 2.1 ± 1.4 N) while allowing an efficient transmission of pulsatile flow. The compliance of the grafts can be tuned by varying the thickness of the PLC layer. *In vitro*, these vascular substitutes promote the formation of an endothelial cell layer, which should increase the hemocompatibility of the grafts (Fernández-Colino et al., 2019) (Figure 4).

In 2015, Gonzalez de Torre et al. developed a system for hiding bare metal stents (BMS) from the immune system by embedding them in ELR-based hydrogels. The idea involved designing an endovascular device with high mechanical stability and physiological hemocompatibility that could act as a physical barrier for the atherosclerotic plaque. These devices were created by placing a BMS in an appropriate tubular mold and producing a chemically cross-linked hydrogel containing the stent by injecting a mixture of two different solutions containing complementary reacting ELRs. ELRs with different bioactivities (RGD, REDV and ELRs lacking any bioactive sequence) were used in order to evaluate the thrombogenicity and endothelization capacity of the finale ELR-biostents. The lowest platelet adhesion and lowest endothelial cell recruiting capacity were obtained using ELRs lacking a bioactive sequence. ELR-biostents containing REDV and RGD sequences showed a very low platelet adhesion and a good ability to trigger the formation of a continuous endothelial cell layer on the lumen of the ELR-biostent (De Torre et al., 2015).

**Figure 5:**
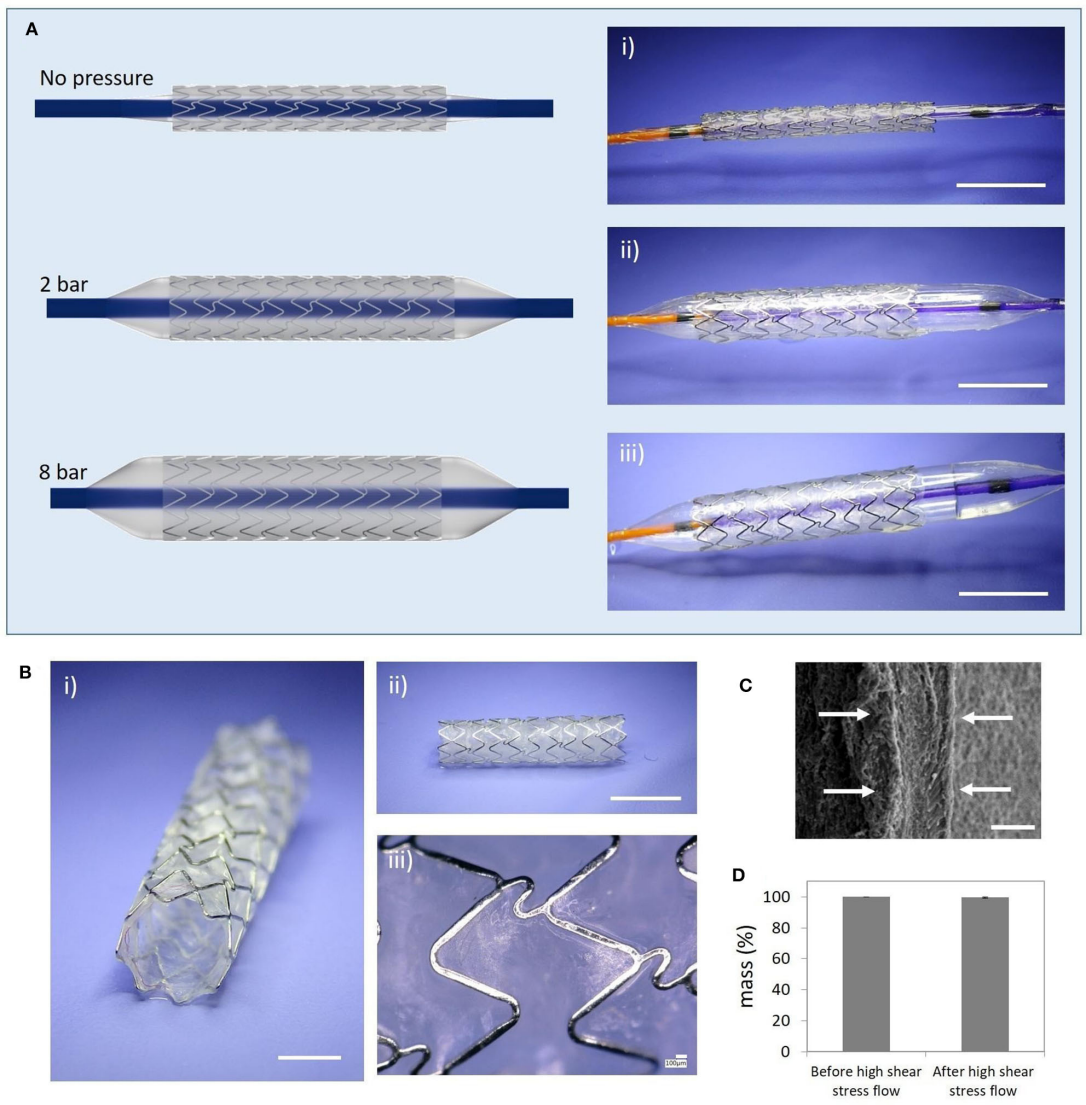
Simulated *in vitro* ELR-covered stent delivery procedure and mechanical stability. **(A)** Balloon-expansion of the ELR-covered stent; (i) initial state before starting the expansion (stent diameter: 1.8 mm); (ii) intermediate state (balloon pressure 2 bar); (iii) final state (balloon pressure: 8 bar; stent diameter: 3.4 mm); **(B)** Deployed ELR-covered stent; (i) semi-frontal image; (ii) lateral image; (iii) detailed view of the ELR membrane; **(C)** SEM image showing the thickness of the layer (white arrows, ~30 μm); **(D)** Assessment of the dry-weight of the covered stent before and after high shear stress flow. Scale bars: **(A)** (i–iii) 5 mm; **(B)** (i) 2 mm, (ii) 5 mm, (iii) 100 μm; **(C)** 20 μm. Reproduced with permission from Fernández-Colino et al. (2019).

Fernandez-Colino et al. have continued the approach of covering stents with ELRs. In 2019, this group published a study in which they developed a coronary stent covered with ELRs using a clickable layer-by-layer (LbL) technique. These coated coronary stents were tested *in vitro* under simulated implantation conditions, demonstrating an excellent elastic performance of the ELR-based coating. Moreover, these devices proved to be mechanically stable under high-flow conditions *in vitro*. From a biological point of view, only minimal platelet adhesion/activation was observed after *in vitro* blood exposure in a Chandler loop. Human endothelial progenitor cells were able to form a continuous and confluent endothelial layer over the ELR membrane covering the coronary stent (Figure 5).

These latter two approaches based on covering BMS with ELRs seem to be promising alternatives for reducing the risk of re-stenosis and thrombosis after stent implantation and should be tested *in vivo* to confirm the potential non-inflammatory nature of the ELR-covered stents.

## Heart Valves

**Figure 6:**
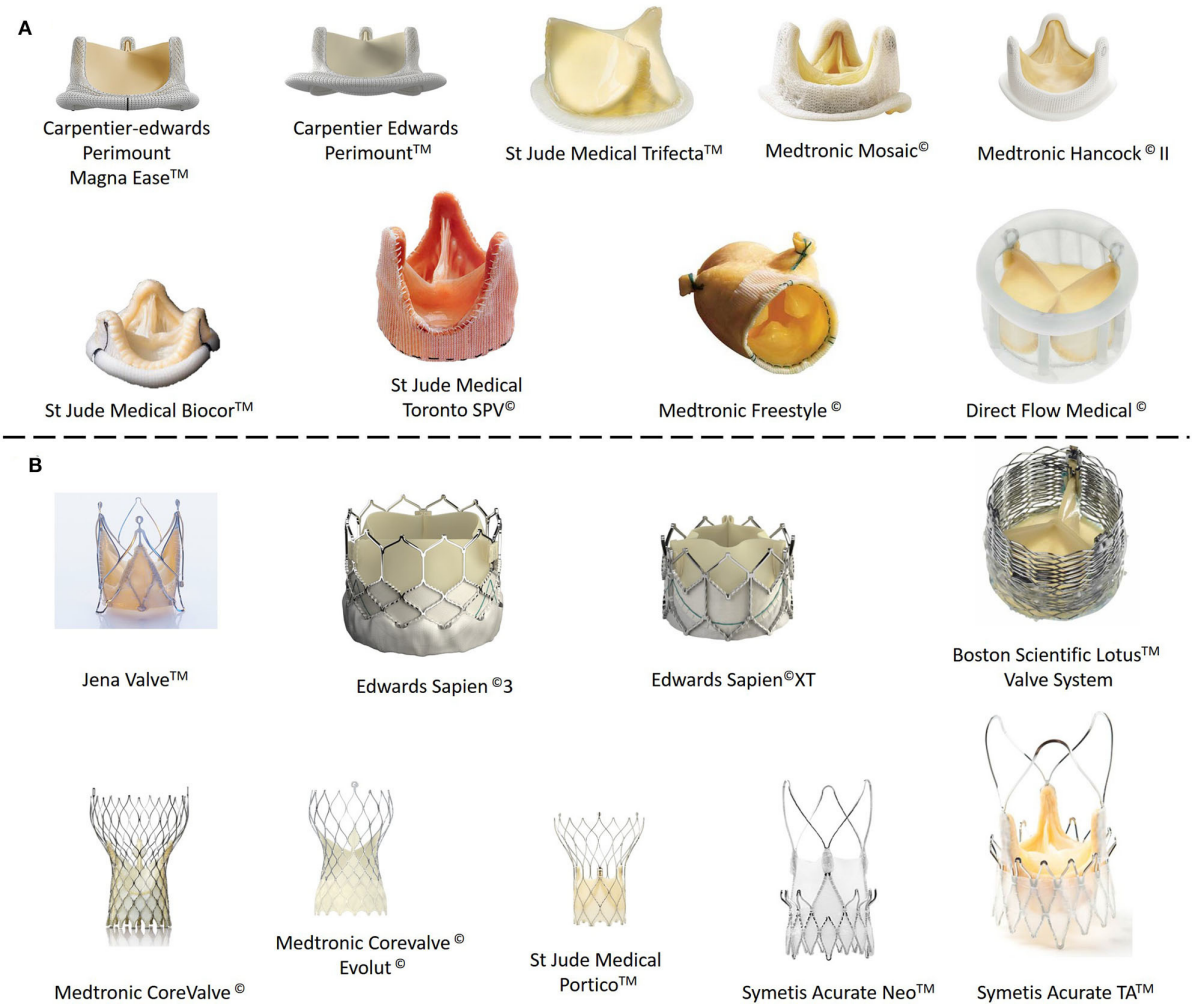
Commercial biological (ovine or porcine tissue) heart valve prostheses. **(A)** Stentless prostheses. **(B)** Heart valve prostheses with stent support.

In the previous few pages we have reviewed the state-of-the-art as regards the tissue engineering approaches that are being explored by scientists for cardiac muscle and the vascular network that supplies it with nutrients and oxygen. However, the heart has another internal structure, namely heart valves (aortic and mitral valves), that is frequently affected by genetic malformations, stenosis or simply failure. Aortic stenosis (AS) is the most common type of aortic valve disease (AVD), with a prevalence of around 0.5% in the general population and 5% among people aged over 75 years. AS is a severe condition that reduces life expectancy to <10 years if untreated, with half of patients dying within only 2–3 years. Calcification of the aortic valve is characterized by fibro-calcific remodeling of the valve leaflets, which lose elasticity and mobility and leads, in severe cases, to blood obstruction. Currently available drugs are not effective at stopping or reverting the process of native aortic valve degeneration (Lindman et al., 2016; Baumgartner et al., 2017; Musumeci et al., 2018). As such, existing solutions are based on surgical repair, reconstruction and replacement. Since introduction of the first artificial aortic valve by Hufnagel et al. (1954), the field of prosthetic heart valves has experienced a continuous evolution, with improvement of the design and performance of both mechanical and biological and tissue-engineered heart valves. However, both kinds of prosthetic heart valve (mechanical and biological) have important drawbacks. Thus, although mechanical valves made from different materials (stainless steel, pyrolitic carbon, or ceramic) and with different architectures (caged-ball, monoleaflet, and bileaflet) were the most common type of implanted prosthesis for many years, the need for lifelong oral anticoagulation medication to prevent thrombus formation swiftly highlighted the need for biological prostheses (Arsalan and Walther, 2016; Søndergaard et al., 2018). Currently, some 87% of aortic valves that require replacement are substituted by bioprostheses, which are mainly obtained from porcine and bovine tissue (Beckmann et al., 2015). As in the case of vascular grafts, one of the first approaches for obtaining bioscaffolds for the creation of heart valve bioprostheses was to decellularize natural heart valves from human donors or from animals (Tedder et al., 2009). The latter are the most widely used due to the high availability of these tissues compared with those from human donors. Similarly, several physical, chemical and biological techniques can be used to obtain acellular scaffolds (Crapo et al., 2011). Bioprostheses are hemodynamically superior to mechanical valves, and amongst biological valves, bovine ones seem to have better hemodynamic performance than porcine valves, thereby reducing the structural valve deterioration (SVD) that usually appears at around 10 years post-implantation (Fries et al., 2000; Lehmann et al., 2007, 2011; Yap et al., 2013). As noted above, although bioprostheses have some advantages with respect to mechanical valves, they are not exempt from problems. In addition to SVD, which is mainly caused by calcification in the commissural and basal areas of the cusps, tearing of the leaflets, calcific degeneration, endocarditis, pannus (abnormal thickening of the valve tissue, mainly in the cusps) and thrombus are the main complications after implantation of a bioprosthesis (Musumeci et al., 2018). The two leading acellular TEHVs available on the market, namely Cryolife's SynerGraft® and AutoTissue GmbH's Matrix P plus N™, both exhibit high variability as regards the results post-implantation and can provoke strong inflammatory responses, conduit failure, pseudoaneurysms, and allograft dissection, with the need for graft replacement, and a pulmonary artery pressure similar to native valves in healthy subjects (Simon et al., 2003; Brown et al., 2011; Perri et al., 2012; Voges et al., 2013). All these factors mean that these artificial valves do not match the behavior and durability of their natural counterparts, with a new valve replacement being necessary 10–15 years after the first prosthetic valve implantation (Yacoub and Takkenberg, 2005) (Figure 6).

A completely different approach involves building a new valve by combining a degradable biomaterial with cells to create a living implant. This tissue-engineering approach is currently being studied by several research groups to obtain functional heart valves that can undergo cell remodeling *in situ* and even grow (in the case of pediatric patients). The base concept of heart valve tissue engineering (HVTE) relies on degradable artificial heart valve shaped scaffolds that are able to evolve into viable living tissues with growth potential following a “normal” inflammatory response. This inflammatory response should start just after implantation of the scaffold and colonization by immune cells recruited from the blood stream, followed by the arrival of macrophages, lymphocytes, and progenitor cells from surrounding tissues, attracted by the cytokines secreted by the immune cells (Bouten et al., 2018). Finally, the scaffolds should be remodeled or even completely degraded by the cells while new endogenous extracellular matrix is produced, replacing the scaffold structure and producing a completely functional living heart valve.

Many biopolymers have been explored as biomaterials for TEHVs. These biopolymers can be classified into two main families: synthetic biopolymers and biological protein-based polymers. Synthetic biopolymers, such as PGA, PLA, PCL, and poly(4-hydroxybutyrate) (P4HB), offer the possibility to be tailored to provide precise control over the mechanical and chemical properties and degradation rate (Filová et al., 2009; Pérez et al., 2013). Some limitations of these synthetic biomaterials include their initial stiffness and rigidity, which could lead to stenosis of the implant. Inappropriate degradation rates (faster or slower than needed) can lead to structural incompetency or chronic inflammation, which produces fibrosis, respectively (Sodian et al., 2000; Ghanbari et al., 2009). Biomaterials based on biological polymers, mainly proteins, such as collagen and fibrin, have been extensively used to design and create TEHVs. Collagen is the most abundant component of the fibrosa layer of natural aortic valves and fibrinogen—a fibrin precursor—can be obtained from the patient's own blood and used to generate autologous, molded fibrin heart valves.

The mechanical properties of collagen-based TEHVs were explored by Shy et al. in a study in which a mixture of solubilized collagen and neonatal rat aortic smooth muscle cells (NRASMC) was used to create mitral heart valves and their chordae. Constructs were stimulated in a specially designed bioreactor and the failure stress values (200 kPa) recorded and compared with non-stimulated samples (100 kPa). Biological parameters, such as the collagen and DNA content of the samples pointed to an increase in extracellular matrix deposition in stimulated samples (Shi et al., 2005). In a similar study, Chen et al. examined the impact of the composition of the collagen-based scaffolds used for TEHVs by evaluating the microstructure and, biological and mechanical properties of scaffolds made from mixtures of collagen, elastin and gycosaminoglycans. Depending on the composition, the tensile, compressive, and bending moduliranged from 39.8 ± 8.8, 10.2 ± 1.6, and 25.4 ± 5.1 kPa to 344.6 ± 42.6, 28.3 ± 4.6, and 78.3 ± 9.7 kPa, respectively. The cytocompatibility of the scaffolds was evaluated by culturing cardiosphere-derived cells (CDCs), which are able to differentiate into cells of the three cardiac lineages and are derived from the endogenous stem cell population in adults. These cells showed good cell proliferation on the collagen-based scaffolds (Chen, 2012).

The research groups of Tranquillo and Jockenhoevel have focused their efforts on designing and producing fibrin-based heart valves, using different approaches to obtain TEHVs that mimic the behavior of their natural counterparts (Jockenhoevel et al., 2001; Weber et al., 2014; Reimer et al., 2017). These TEHVs are based on a tubular leaflet design that can eventually incorporate a textile reinforcement to increase the mechanical properties of the fibrin gels used as biomaterials. These approaches comprise the use of several types of cell sources, such as ovine dermal fibroblasts, vascular cells isolated from ovine umbilical arteries or human vascular cells, amongst others. The resulting fibrin-based TEHVs exhibited similar mechanical properties, with a modulus about 4 MPa and performances similar to the native versions (Moreira et al., 2016; Reimer et al., 2017). Although fibrin-based TEHVs showed excellent hemodynamic performance *in vitro* and *in vivo* (Reimer et al., 2015), most of them exhibited shrinkage of the leaflets, which produces regurgitation and leads to their failure (Flanagan et al., 2009; Gottlieb et al., 2010; Syedain et al., 2011; Driessen-Mol et al., 2014; Reimer et al., 2017). One of the main causes of this shortening of the leaflets is the very low production of elastin required to provide elasticity in the leaflets in order to complete coaptation of the leaflet and closure of the valve and avoid undesirable regurgitation events. Moreover, this low elastin production and high extracellular matrix secretion, mainly collagen, by the cells colonizing the scaffolds produces a stiffening of the leaflets, thus enhancing their shrinkage and leading to a non-functional TEHV. These findings suggest that the role of elastin in the proper performance of heart valves is crucial. The aortic valve cusp is a three-layered structure (fibrosa, ventricularis, and spongiosa) with different proportions of collagen and elastin but with an average of about 13% elastin in healthy adults. During diastolic loading, the cusps extend with a strain of more than 50%, then recoil elastically. As collagen is not a highly elastic protein, this elastic behavior must be due to the presence of elastin. Taking into account this low percentage of elastin and its high importance in the elasticity of the leaflets, it is clear that the arrangement of the elastin fibers and their interaction with collagen must play a paramount role. Scott and Vesely suggested an interaction between collagen and elastin that could explain the mechanical behavior of the leaflets in aortic valves. This mechanism proposes that elastin may act as a return spring by establishing connections between different collagen fibers or between different regions of the same collagen fiber. Once the collagen fibers are stretched, they take up a great proportion of the load. The elastin in the aortic valve then restores the geometry of the collagen fiber to its original conformation between successive loading cycles and, consequently, is essential for proper functioning of the valve (Scott and Vesely, 1995). This close interaction between collagen and elastin is reflected in the mechanical properties exhibited by heart valves (Vesely, 1997). The authors conclude by introducing the idea that any biomaterial intended to emulate the micromechanisms of heart valves should take into account the close relationship between collagen and elastin.

**Figure 7:**
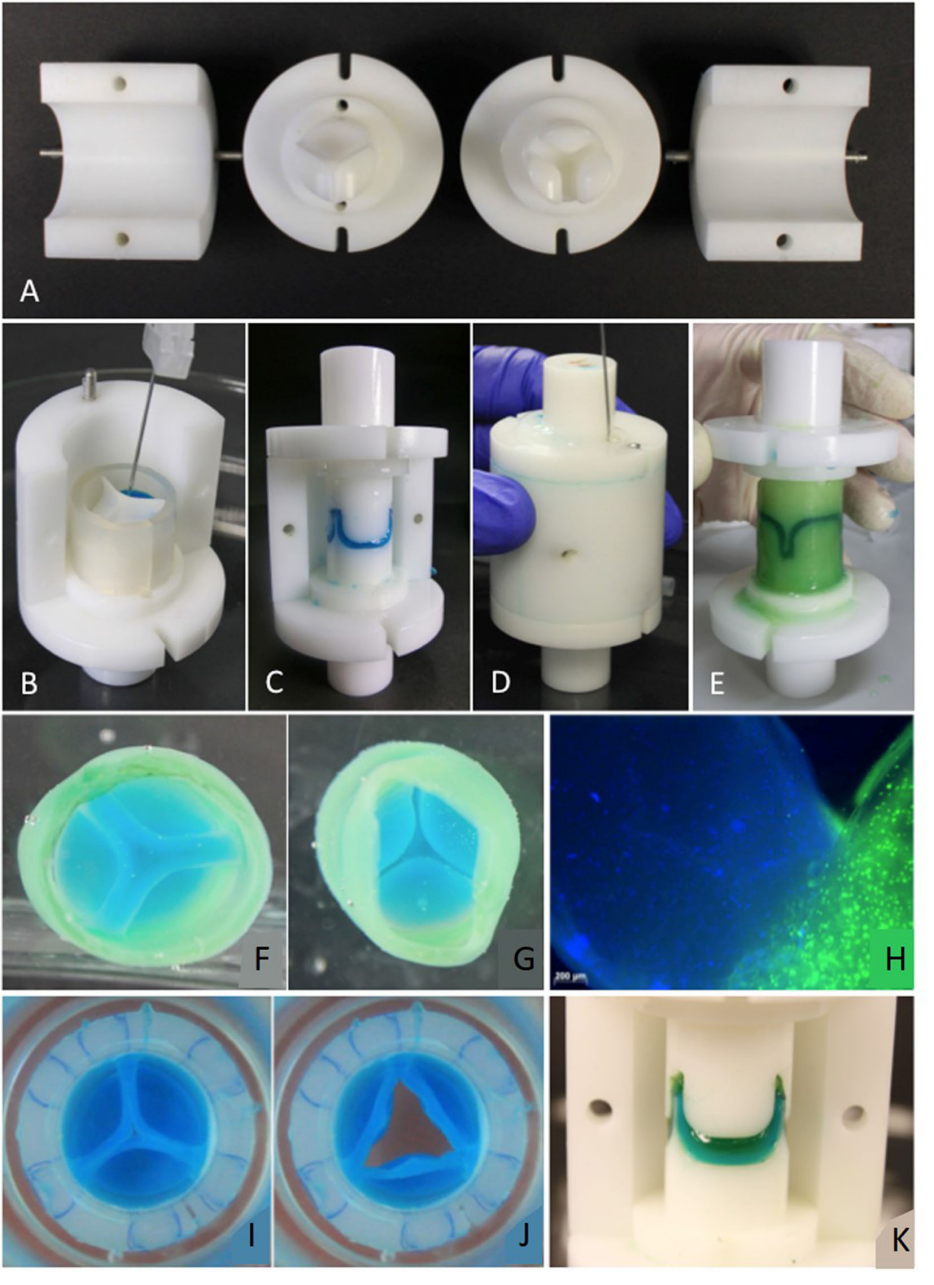
**(A)** Mold parts: Ventricular and vascular stamp and shells. Molding procedure (Valve molded with colored fibrin gel to prove feasibility of molding design): **(B)** The components of the leaflets are inserted in the mold. **(C)** The leaflets are molded by placing the vascular stamp on top. Afterwards, the silicone ring is removed. **(D)** The components for the wall are inserted into the mold with a needle. **(E)** For demolding, the shells and then the stamps are removed carefully. **(F)** View from “vascular” side. **(G)** View from “ventricular” side. **(H)** Commissure area of a TEHV molded with live stained cells. The nuclei in the leaflet are stained blue with Hoechst 33258, the cytoplasm of cells in the valvular wall green with Calcein AM. **(I,J)** TEHV with leaflets made of an ELR-fibrin hybrid gel and the wall made of fibrin. **(K)** Leaflets made of two layers of fibrin (first layer blue, second layer green). Reproduced from Weber et al. (2014).

To the best of our knowledge, there is no approach to building THEVs based exclusively on elastin or elastin-like biomaterials. Taking into account the findings regarding the composition of natural valves and the approaches based on collagen or fibrin mentioned above, it seems that the path to functional TEHVs is likely to involve the use of hybrid materials that combine the mechanical and biological properties of their individual components. In this sense, hybrid ELR-fibrin gels have been created and characterized mechanically and biologically. These ELR-FGs showed better mechanical properties than the pure fibrin gels used to create TEHVs as they combine the elastic properties of ELRs with the well-known cell-affinity of fibrin gels. Moreover, these ELR-FGs served as excellent materials to fabricate semilunar heart valves that could be colonized by ovine umbilical smooth muscle cells (OUSMCs). These cells were able to create their own extracellular matrix, forming collagen I and III as well as alpha-smooth muscle actin with some degree of alignment in TEHVs cultured *in vitro* for 21 days (Gonzalez De Torre et al., 2016). Following this hybrid ELR-FG based approach, Webber et al. developed TEHVs using a multi-step injection molding system, thus paving the way to the creation of TEHVs with a heterogeneous composition from a cell and scaffold material point of view. This system was intended to create TEHVs with different compositions for the walls and leaflets of the engineered valves without the need for suturing or gluing the different materials, thus ensuring that the leaflets are firmly attached to the wall of the TEHV. The functionality and integrity of the engineered valves were tested *in vitro*, with and without cells and with continuous stimulation for 2 weeks. Using this multi-step molding technique, different cell lines were embedded in different regions of the TEHVs and proliferated separately, generating their own ECM, which means that both the material arrangement and cell composition can be controlled and precisely placed in the desired region of the TEHV (Figure 7).

## Conclusion and Future Perspectives

In light of the above, it can be deduced that elastin is a crucial component of the extracellular matrix in cardiovascular tissues, although it is not the major component in terms of dry weight. Despite its paramount role in cardiac tissues, biomaterials based on elastin or elastin-derived materials have mostly been used to create artificial vascular grafts. Elastin-derived materials have been processed, either alone or mixed with others, in several ways, including electrospinning and molding, to obtain artificial tubular constructs that can act as blood vessel substitutes. These different approaches have shown that elastin-derived biomaterials are non-thrombogenic and cell-friendly materials that are close to reaching preclinical and clinical stages after promising *in vivo* results. However, and although the research in ELRs started some decades ago, it can be seen that most of the studies and works in cardiovascular regeneration summarized in this review are from the last 5–10 years, a boom period in which we still are, and which is a short time for reaching preclinical or clinical trials.

Scaffolds for cardiac muscle regeneration is the second area of cardiovascular tissue engineering in which elastin-based materials have been applied. Recently, ELR-based scaffolds have started to gain importance as they can induce the rapid angiogenesis needed for cardiac muscle regeneration thanks to the introduction of angiogenic and specific protease sensitiveness. However, only a limited number of examples in which elastin-based scaffolds are used directly as cardiac patches can be found in the literature, and only a few of these have been applied *in vivo*.

Heart valves are the third big area explored in this review as regards cardiovascular tissue regeneration. As has been discussed, elastin plays a crucial role in the proper functioning of HVs and a lack therefore is possibly the main reason for shortening of the leaflets in TEHVs. Despite its obvious importance in this kind of structure, very few approaches have used biomaterials based on elastin or elastin-like materials.

It is worth pointing out that, under our knowledge, no clinical trials have been done with ELRs-based scaffolds for cardiac tissue regeneration although some preclinical test (Ibáñez-Fonseca et al., 2018) and patent based on ELRs have been registered (EP3124495A1; US9328154B2; WO/2010/092224; WO/2012/168532 and others). There are several reasons that could explain this fact but all of them fall under the speculation. The first one is the novelty of these biomaterials. It is important to understand that ELRs have gained importance in the last two decades, attending to the number of references that can be found in literature, and this is a short time for a biomaterial to reach clinical trials. The tailored design of these molecules is a double-edged sword, in one side it has enormous potential and benefits as for instance to design and precisely incorporate the desired bioactive sequences in the backbone of the polymer. However, in the other side, the possibility to create a huge variety of ELRs lead to another big challenge from the regulatory point of view. Many of the ELRs are more than mere polymeric materials without any bioactive property by themselves. They are more than scaffolds that offer support and mechanical properties; they can be designed to develop an active role during the regeneration process. This complexity in their design and functionality is transferred to regulatory aspects; actually, it is not easy to elucidate if these ELRs based materials should be classified as drugs, medical devices or even as advanced therapy medical products (if they are used with cells embedded).

Many improvements have been achieved in the field of tissue engineering for cardiovascular applications, including improvements in our understanding of the macro- and micromechanical performance of these tissues, improvements in the cell-ECM interaction and cell function, improvements in new biomaterials, improvements in processing technology, and *in vitro* experiments. However, unfortunately, these improvements are yet to produce a completely functional bioscaffold that could be implanted into patients and could mean actual regeneration of the damaged tissue. As such, there is still room for further improvements in the cardiovascular field, especially as regards biomaterials, in which elastin-derived materials should become more important and attract the attention of research groups focused on cardiovascular tissue engineering.

## Author Contributions

IG has written the review. J-CR-C and MA have helped in reviewing the manuscript.

## Conflict of Interest

The authors declare that the research was conducted in the absence of any commercial or financial relationships that could be construed as a potential conflict of interest.
